# Sick-listed workers’ experiences with motivational interviewing in the return to work process: a qualitative interview study

**DOI:** 10.1186/s12889-020-8382-9

**Published:** 2020-02-28

**Authors:** Vegard Stolsmo Foldal, Martin Inge Standal, Lene Aasdahl, Roger Hagen, Gunnhild Bagøien, Egil Andreas Fors, Roar Johnsen, Marit Solbjør

**Affiliations:** 1 Department of Public Health and Nursing, Faculty of Medicine and Health Science, Norwegian University of Science and Technology, Postboks 8905, MTFS, 7491 Trondheim, Norway; 2 Department of Psychology, Faculty of Social and Educational Sciences, Norwegian University of Science and Technology, Trondheim, Norway; 3 Unicare Helsefort Rehabilitation Centre, Rissa, Norway; 4 Tiller Community Mental Health Centre, Division of Psychiatry, St. Olavs Hospital, Trondheim University Hospital, Trondheim, Norway

**Keywords:** Motivational interviewing, Return to work, Sick leave, Self-efficacy, Professional–patient relationship, Qualitative research

## Abstract

**Background:**

When returning to work after being on long-term sick leave, individuals may experience varying levels of motivation and self-efficacy. Motivational interviewing (MI) is a counseling style that aims to increase motivation towards change, and it may be useful in the return to work (RTW) process. The aim of this study was to explore sick-listed workers’ experiences with MI in the RTW process.

**Methods:**

This qualitative study was part of a randomized controlled trial evaluating the effects of MI on the RTW process, and it was administered by caseworkers at the Norwegian Labor and Welfare Administration. Sixteen sick-listed individuals, aged 33–60, participated in semi-structured interviews. All had a sick leave status of 50–100% for at least 8 weeks when interviewed and all had completed 2 MI sessions. The data was analyzed with systematic text condensation.

**Results:**

Participants’ experiences of the MI sessions were categorized into three themes: (1) relationship with the MI caseworker, (2) normalizing sick leave, and (3) adjusting RTW strategies. The MI sessions were experienced as a positive encounter due to the supportive relationship that was built between the MI caseworker and the sick-listed worker. Being sick listed led to feelings of guilt and stigmatization, but acceptance and support from the MI caseworkers helped normalize the situation for the sick-listed workers. Furthermore, MI sessions allowed for personalized feedback and discussions on adjustments to their RTW strategies.

**Conclusion:**

Sick-listed workers experienced MI as positive due to the good relationship that developed with the MI caseworker, how this normalized sick leave, and the help they received with adjusting their RTW strategies. Professionals working with individuals attempting to RTW may benefit from using MI as a method for helping sick-listed workers to RTW.

**Trial registration:**

ClinicalTrials.gov: NCT03212118 (registered July 11, 2017).

## Background

Work is central to an individual’s health, identity, social role, and status [1]. Long-term sickness absence is challenging for the individual, their employer, and society [2]. Despite various targeted efforts to increase return to work (RTW), there are no conclusive results on what is an effective RTW approach [3–5]. However, it has been suggested that social support, motivation, and self-efficacy play a central role in the RTW process [6–8].

Planning how and when to RTW after long-term sick leave is difficult for the individual worker, and adhering to a RTW plan may also be challenging [9]. Support and encouragement from RTW professionals, such as social insurance caseworkers and health care professionals, may empower and enable the sick-listed worker to RTW [10]. Two important predictors for RTW are social support and self-efficacy [11, 12]. Self-efficacy is the belief in ones’ ability to achieve a given goal or task [13]. Support from RTW professionals may positively affect the sick-listed workers’ self-efficacy and help them achieve their RTW goals [14]. This suggests that focusing on sick-listed workers’ self-efficacy and establishing a positive and respectful relationship between the sick-listed worker and the RTW professional may be a successful approach for improving RTW [14].

Motivational interviewing (MI) has been suggested as a possible approach to promote these factors in a RTW process [15]. MI is a client-centered and directive counseling method aimed to facilitate intentional and behavioral change. The method was first developed for treating alcohol abuse [16], and it was later shown to be effective in various clinical settings and in short interventions [17–20]. MI has been found to be effective by only single sessions [21] and even in small doses of 15 min [20], and can therefore be offered as an early low-intensity intervention. In MI, it is essential that the counselor seek to establish a collaborative partnership with the client and use communication skills to strengthen the client’s motivation for change [16]. In Norway, the Norwegian Labor and Welfare Administration (NAV) recommends that their caseworkers apply MI when counseling sick-listed workers in the RTW process [22]. Only a few studies have evaluated the effect of MI on RTW for sick-listed workers, and evidence of the method’s efficacy as a RTW intervention is lacking [15, 23]. However, a recent study found that the use of MI led to more sustainable RTW compared to traditional rehabilitation for patients with musculoskeletal complaints [24]. Moreover, a Swedish study found that unemployed long-term sick-listed individuals experienced their encounters with RTW professionals using MI as positive [14]. These findings suggest that MI may be useful in a RTW context. However, research on how sick-listed workers experience MI counseling in a RTW context and how this affects their RTW process is lacking. Therefore, the aim of this study was to explore sick-listed workers’ experiences with MI in the RTW process.

## Methods

The present study was based on 16 semi-structured individual interviews with sick-listed workers enrolled in a randomized controlled trial (RCT) [25]. This approach was chosen to explore sick-listed workers’ experiences with MI in the RTW process.

### The randomized controlled trial (RCT)

The overall RCT in which this qualitative study is nested, aims to evaluate MI as an instrument for caseworkers at NAV in facilitating RTW for sick-listed workers. The RCT has a three-armed group design. Eligible participants for the RCT were all sick-listed workers, 18–60 years old, living in central Norway, with unselected diagnoses. Their sick-leave status at the time of inclusion in the RCT had to be 50–100% for at least 8 weeks. Exclusion criteria were pregnancy-related sick-leave and unemployment. All participants randomized to the MI intervention group were offered one MI session at 14 and one MI session at 16 weeks of sick leave, in addition to standard NAV sickness absence follow-up. Each MI session had a maximum length of 60 min. Having two 60-min sessions were considered to allow enough time and follow-up to engage in change, and at the same time be considered a brief intervention [25]. The findings from the RCT and the present interview study will be independently reported and not systematically matched in the present article.

### The Norwegian welfare system and sickness absence follow-up

Compared to other OECD countries, Norway has a high sickness absence rate [26], and this has been stable the last decade, with a current sickness absence of 5.9% [27]. About 85% of individuals on sick leave RTW before 12 weeks of sick leave, whereas only 7.4% RTW between 12 and 26 weeks of sick leave [28]. In Norway, employees are entitled to full wage benefits in the case of sickness absence, from the first day of absence to a maximum period of 52 weeks. During the first 16 working days, the employer is responsible for the payment, while the rest is paid for by the National Insurance Scheme through NAV [29]. The employer must initiate a follow-up plan in cooperation with the employee before the end of fourth week of sick leave and is responsible for arranging a meeting with the sick-listed worker within the seventh week of absence, including other stakeholders, if relevant. If the employer does not initiate a follow-up plan, NAV has no possibility to sanction the employer. If the employer facilitates work-related activities, the sick-listed worker is expected to participate. If the employee does not begin work-related activities within 8 weeks, an expanded medical certificate is required to document that the employee has significant medical problems preventing them from participating in work-related activities. Sick-listed workers who do not engage in work-related activities, without a medically certified reason, can be sanctioned by NAV with reductions in sickness benefit payments. NAV is responsible to arrange a meeting which includes the employer and the sick-listed worker, around and or no later than, 26 weeks of sick leave. The attendance of the sick-listed employee’s general practitioner is optional. However, the general practitioner is obligated to attend if NAV deems it necessary for the coordination of the RTW process. An additional meeting is held if one or more of the stakeholders find it necessary. The sick-listed worker may also ask for a meeting with NAV to coordinate a plan for RTW outside this schedule [29].

### Motivational interviewing (MI) sessions

In addition to the usual follow-up by NAV, two extra MI sessions were offered to the sick-listed workers by a NAV caseworker after being on sick leave for 14–16 weeks [25]. The sick-listed workers were informed that the MI sessions were part of a research project and did not affect their rights or obligations as sick listed. However, they did not receive information from the caseworker that they would be using the MI counseling style.

In the MI sessions, the caseworker tried to engage the sick-listed worker in a collaborative relationship by using person-centered communication skills. During the first session, the sick-listed worker was offered to choose the agenda from a written menu describing different areas of life that the situation as sick listed could affect. These areas could also affect the RTW process. To adjust the intervention accordingly, the client’s stage of motivation [30] for RTW change was assessed. The client’s own motivations for RTW change were explored and focused on, as well as the sick-listed confidence in RTW. In the second session, the caseworker aimed to map the sick-listed individual’s current work tasks and earlier attempts of RTW. Information exchange of available support from NAV during the RTW process was included. The sick-listed worker’s self-efficacy was assessed, and his or her future work goals were explored. The sick-listed worker’s readiness for RTW change was assessed, and a written action plan was developed if the sick-listed worker was ready for RTW change. Whether or not a written RTW plan was made, the caseworker provided the sick-listed worker with a written summary of the two MI sessions [24]. At the end of the first MI session, they were informed that a written summary remained available for the sick-listed and assigned caseworkers, which is a standard procedure in the sickness absence follow-up at NAV.

Four NAV caseworkers offered the MI intervention, in addition to handling their usual workload in the sickness absence follow-up at NAV. The caseworkers were trained to develop the necessary MI skills, consisting of three-hour sessions twice a week for 6 months prior to recruitment into the RCT. To ensure that the sessions consisted of valid MI content, the caseworker used a standardized MI guideline developed by the research group. Three MI experts offered the training, and the caseworkers were supervised, including use of audiotapes in order to maintain and further develop their MI skills [25].

### Recruitment and participants

To participate in the current qualitative interview study, the sick-listed worker had to have already completed two MI sessions as part of the RCT. All study participants who had completed two MI sessions between November 2018 and January 2019 were identified by NAV (*n* = 29). Contact information (cell phone number) of these individuals was forwarded to the researchers. One of the authors (VSF or MIS) called these participants to invite them to take part in the research interview. Of the 29 individuals who were invited to participate in the interview study, 13 did not answer, declined the invitation, or did not show up at the interview. Sixteen individuals, three men and thirteen women, participated in the interviews. They were aged 33–60 years and had a sick leave status varying from 50 to 100%, except one participant who since inclusion to the RCT had been graded to 40% sick leave at the time of the interview study (see Table 1 for participants’ descriptive information). All information in the present study was supplied from the participants during the interviews, and no other information from neither NAV or the RCT was used or matched into the current interview study.

**Table 1:** Participants’ descriptive information

	*n*
Gender	
Male	3
Female	13
Age	
33–39	2
40–49	4
50–59	9
60–64	1
Self-reported reason for sick leave	
Common mental health disorder	8
Musculoskeletal disorder	5
Other	3
Education level	
High school	2
College/university up to 3 years	4
University more than 3 years	8
Working sector	
Public	8
Private	8

*Education is described as the participant’s highest completed education (higher education is at the university/college level). Level of education is missing for two participants*

### Data collection

To explore the participants’ experiences, semi-structured individual interviews were performed, which allowed the participants to provide in-depth descriptions of their experiences and it provided an opportunity for follow-up questions from the researcher. The interviews were based on an interview guide, with five main questions concerning their experiences during sick leave, the RTW process, and the first and second MI sessions, as well as whether these sessions led to any changes during their RTW process (see Additional file 1: Interview guide). The interviews were conducted between November 2018 and January 2019. Ten of the interviews were conducted by the first author, VSF, and six interviews were conducted by the author MIS. The interviews lasted from 25 to 66 min (mean time of 42.5 min). Two of the interviews were considerably shorter than the other interviews (lasting 25 and 27 min), and two interviews were considerably longer (64 and 66 min). The interviews were audio recorded and transcribed verbatim. Due to the semi-structured nature of the interviews, the experiences shared by the participants where not strictly limited to each question asked. Some main questions were answered more briefly, and other questions answered more in-depth, with accompanying probing questions. All participants were willing to share their stories and experiences about their RTW process, although the level of detail in their descriptions and time spent on each main question varied. Some interviewees cried during the interview when telling their story, whereas a few of them were reticent in their descriptions.

Malterud et al. [31] suggested a guide for determining an adequate sample size to obtain information power in qualitative studies based on aim of the study, sample specificity, theory, dialogue quality, and analytical strategy. Inspired by this, the desired number of interviews was evaluated before conducting any interviews, and during primary analysis after nine and sixteen interviews. We aimed to collect a variety of experiences from the MI sessions. Therefore, we allowed for different participant characteristics without defining a diagnostic specific sample. The quality of the dialogue and the collected data were considered satisfactory enough for uncovering the varying experiences of the participants. Between the ninth and sixteenth interview, we did not find more variety in experiences, and the data were judged satisfactory saturated for our purpose.

### Data analysis

The data analysis was based on systematic text condensation, which was originally inspired by descriptive phenomenology [32]. Systematic text condensation is a four-step descriptive analytical process with explorative ambitions to describe the experience of the participant as they express them. The first step of the analysis involves reading the data as a whole to get an overall impression of the data and to identify possible themes [32]. The transcripts of the first four interviews were read before further interviews were carried out, which allowed us to recognize preliminary themes. After this reading, minor adaptations to the interview guide were made in order to improve the wording of the questions and to add some specific follow-up questions regarding the MI sessions. This process was repeated after the ninth and sixteenth interview. The second step of the analysis was to identify and sort meaning units [32]. In the preliminary analysis, meaning units were coded and sorted into seven themes; Human relations (1), Being acknowledged (2), Orientation towards RTW (3), Practical information (4), Support towards RTW (5), Shame of being sick-listed (6), and Legitimizing sick leave (7). After all interviews were finished, the number of distinct themes was reduced to three main themes, which covered the data from the previous seven overlapping themes. The first two preliminary themes were reduced into one distinct theme, whereas preliminary themes 3, 4 and 5 were grouped into the second final theme. Lastly, preliminary themes 6 and 7 was reduced to the third and final theme. The third step of the analysis was what Malterud [32] termed “condensation,” which involves decontextualizing the meaning units by rewriting them into illustrative quotes. By rewriting the meaning units into first-person narratives, we created a sum of the participants voices regarding the phenomenon that was described in the data. The fourth step of the analysis entailed synthesizing descriptions and concepts by recontextualizing the condensations [32]. The condensations were checked against the “raw” data in the transcripts in order to validate that the findings represented the experiences described by the participants. Then they were recontextualized into themes and validated by all other authors. Finally, the recontextualized meaning and phenomena were written together into an analytical text, as presented in the results. The author VSF read and analyzed all interviews. All other authors read and analyzed two or more interviews each, thus striving for more nuanced perspectives on the analysis and possibly reducing single-researcher preconceptions.

### Ethics

All participants received written and oral information about the study and gave their written consent before the interview started. Participants were informed that participation was voluntary and that they could withdraw from the study at any time. None of the participants opted to do so. The interviewer attempted to be friendly and accommodating during the interviews. All participants were offered a post-interview debriefing, and they were also invited to contact the interviewer if they had any further questions regarding the research, the analysis, or their interviews at a later stage. The study was approved by The Regional Committee for Medical and Health Research Ethics in South East Norway (REK nr 2016/2300).

## Results

The participants had some common features, which were not related to their experiences during the MI sessions, but were instead related to their backgrounds before participating in the sessions. These participants had little or no knowledge about being on sick leave or what it entailed in terms of rights, obligations, and possibilities. Their RTW plans varied in terms of RTW strategies, the involvement of employers and helpers, the level of detail in the plan, and whether it was written down or agreed upon orally. Workplace adaptions were important when describing what could enable them to RTW, while the lack of workplace adaptions was disabling for the RTW process. The participants’ experiences of the MI sessions could be categorized into three themes: (1) relationship with the MI caseworker, (2) normalizing sick leave, and (3) adjusting RTW strategies. The first theme describes how these participants experienced their relationship with the assigned MI caseworker. The second theme is about how the participants discussed their situation as a sick-listed employee with the MI caseworker. The third theme concerns participants’ experiences of the content during the MI sessions.

### Relationship with the MI caseworker

The participants had few expectations regarding the involvement of NAV in their RTW process. They expected that NAV would be absent during their RTW process, at least during the first 6 months of sick leave, and that any activity directed towards them would be about controlling their entitlement to sick-leave benefits. They also expected that NAV would be hard to reach and experienced that receiving messages or letters from NAV was insufficient in terms of motivating the sick-listed worker in their RTW process.

*“Merely receiving a letter from NAV does not feel as if they take an interest in you. If NAV had more contact with people, they would be able to push people in the right direction [back to work]…” – Female (age 60)*

However, when meeting the MI caseworker, their negative expectations about NAV changed. The sick-listed workers experienced a satisfying and supportive relationship with the MI caseworkers, whom they described as skilled, trustworthy, and with a kind but professional appearance.

The MI caseworkers were described as both accommodating and informative; the latter description was due to ability to provide tailored alternatives in RTW strategies for the sick-listed workers. The MI sessions were an arena where they felt acknowledged and cared for. The MI caseworkers asked questions about several aspects of their lives that could be related to their situation as a sick-listed worker, and they appeared attentive when listening. For participants, the personality of their MI caseworker appeared to be matched to their own in terms of sense of humor, expressive communication style, and personal interests. This allowed them to appreciate their relationship with the MI caseworker.

*“….She was accommodating, caring and professional. Yes, I think they had chosen the right person for me there.” – Female (age 54)*

Having a face-to-face encounter with a MI caseworker was emphasized as important to the sick-listed workers. Not only did they receive support from the MI caseworker, but they also appreciated the MI caseworker’s ability to enable and motivate them. Sometimes, the MI caseworker lacked expert knowledge about the participant’s type of work, but by being curious and interested in the story of the sick-listed worker, the MI caseworkers appeared to quickly comprehend their situation at work.

### Normalizing sick leave

When faced with questions about the cause of their sick leave from colleagues, their employer, or NAV, the sick-listed workers had to offer a good reason or an explanation for their sick leave. The need for explaining and defending the necessity for sick leave came from the fear of being viewed as someone who did not want to work. This was even more pertinent when being sick listed due to mental disorders. It was easier to talk about and explain a physical illness that was visible to others. One participant that was on sick leave due to a mental disorder had to provide an alternative story to her colleagues.

*“I told someone that I was on sick leave due to back problems, but I’m actually seeing a psychologist. It feels good to talk with someone [psychologist] and clear your head, and I need help doing that.” – Female (age 45)*

Knowing that their sick leave led to higher workloads for their coworkers and extra strain on the employer caused guilt. Even when on graded sick leave, thus relieving the potential strain on both the employer and their coworkers, the guilt remained.

*“It felt like I can’t leave work now, somehow, because I’ve only been here for two, two and a half hours. It felt completely wrong, I felt that it wasn’t okay, I felt guilty about it. I find it difficult to just leave work. I have to be there at least for half a day before I feel like I can go home” – Female (age 33)*

In a difficult situation where participants experienced stigma and guilt, the MI sessions served as an arena for normalizing and providing legitimacy through support from the MI caseworker. The MI caseworker and the sick-listed worker talked through negative thoughts about the stigma and guilt of being sick listed. The MI caseworker explained how common these thoughts were and that the accompanying feelings were normal. Receiving support from a MI caseworker gave legitimacy to the participant’s need for sick leave, and it led to acceptance of this problematic situation. The participants could also discuss concerns about how their illness affected their relationships with their spouses, friends, and children, as well as time for leisure activities. Receiving support from the MI caseworker about all aspects of their RTW process was enabling in terms of transitioning into talking about their RTW strategies. The stigma and guilt that were experienced as barriers in the RTW process were reduced through the dialogue in the MI sessions.

### Adjusting return to work (RTW) strategies

During the MI sessions, the participants received personal feedback about their RTW plan from the MI caseworker, who offered information about their rights and obligations as sick-listed workers, as well as possible future economic benefits from NAV. Since the sick-listed workers had little prior knowledge about what NAV could offer, they experienced gaining insights about available support and measures from the MI caseworker as useful and often incorporated it into their RTW plan.

… *I didn’t know how to relate to it, because I had never been on sick leave before. I knew very little about NAV, you know, I had never been in contact with NAV. So, I didn’t know anything, but I got a lot of useful information from her and about what NAV has to offer.” – Male (age 57)*

The individually tailored information and the support provided by the MI caseworker helped participants to reorient their perception towards workload, work tasks, and working time. The possibility of adjusting their time spent at work and the amount of work they produced was highlighted as new and important information that led to a successful change in their RTW strategy. For one participant, NAV made her aware of the possibility of being present at work full time, while still being on 50% sick leave. This enabled her to work at her own preferred pace and still produce 50% of her expected full-time workload.

*“My plan was to return to work in full-capacity, but I was on 50% graded sick leave at the time, so I worked half days for a while. I didn’t know until the [MI] caseworker told me that it’s not about how many hours you are at work, it’s about how much work you produce. So now, I can be at work an extra hour a day and still have time to do my exercises as a part of my rehabilitation. I don’t have to work more, but I can spend more time on doing it.” – Female (age 52)*

Another important RTW adjustment was in terms of the RTW pace. Whereas some experienced recommendations of a slower approach, others experienced that the MI caseworker endorsed a faster pace. Receiving tailored advice from the MI caseworker on their RTW pace was considered important for a successful RTW process.

*“My plan was to return to work full-time three months after the operation, I thought that I would be ready for it. My [MI] caseworker thought that wasn’t such a good idea, and she suggested that I had a more cautious approach. I now realized that she was completely right, and that it probably was a good reminder for me to listen to my body and take the time I needed to return to work. Retrospectively, If I hadn’t taken it easy, I wouldn’t have handled it [RTW] and probably gotten worse.” – Female (age 47)*

However, if the sick-listed worker was not in need of information or adjustments to RTW, the MI sessions were not experienced as useful. One participant experienced that the MI caseworker challenged his already mapped out RTW plan, which made him reconsider the quality of his original plan. The participants said that they could talk with the MI caseworker about what could happen if they were not able to RTW and what they could do when feeling ambivalent towards their choices during the RTW process. Discussing their ambivalence with the MI caseworker was enabling in terms of their actions towards RTW, where adjustments in RTW strategies were made to varying degrees.

## Discussion

The results from the present study show that the participants experienced a good relationship with the MI caseworkers during the MI sessions. Talking with the MI caseworkers helped the participants normalize their situation as sick-listed workers, reduce the feeling of guilt, and reduce the stigma they experienced. Receiving personal feedback about their RTW plan, either to support their current plan or to reflect upon potential changes to their plan, increased their experienced RTW self-efficacy.

Previous studies have shown that sick-listed workers consider insurance officials to be distant, lacking trust, and questioning the sick-listed workers’ credibility, which may lead to powerlessness during the RTW process [10, 33]. Positive encounters were described in a previous study [33], where the professionals asked what the sick-listed workers wanted and where the participants were treated with respect. In the present study, the sick-listed workers described having a positive and good relationship with the MI caseworkers. This is in line with the findings from a similar study in Sweden, where sickness benefit officials offered a counseling session with unemployed long-term sick-listed workers using a MI approach [14]. Support and encouragement from various professionals may empower and enable the sick-listed worker to RTW [10], and by establishing a good relationship the RTW professional may help the sick-listed worker to overcome obstacles during the RTW process [34]. Despite differences in characteristics, the sick-listed workers in the present study experienced MI as a positive intervention. This may be because the MI sessions were driven by their expressed needs, in combination with creating a good relationship. In MI, the relationship between a counselor (e.g., MI caseworker) and a client (e.g., sick-listed worker) is characterized by acceptance and empathic understanding from the counselor [35]. Forming a good relationship with the client is one of the cornerstones of MI. Having a good relationship can elicit and strengthen the persons’ own reasons for change and their plan of action [35]. Studies have also suggested that the relationship between a counselor and a client is important to the outcome of the treatment [36]. From a RTW professional’s perspective, building an alliance is reported as important to facilitating RTW for sick-listed workers [37].

In the follow-up procedure for sick leave in Norway, caseworkers at the NAV operate as both RTW professionals and as controllers of sickness benefits [38]. This double role can be a conflicting paradox [34] that may hinder a good relationship [10]. In the present study, participants did not report that MI caseworkers controlled their rights to sickness benefits during the MI sessions, which indicates that this was not a barrier to forming a good relationship during the MI sessions. Having positive and supportive encounters with health care personnel and significant others (e.g., NAV caseworker) has been shown to be important in what long-term sick-listed workers experience as successful RTW processes [39]. This is in accordance with findings in the current study, which indicates that using MI may be beneficial for a successful RTW process. Experiences from the Swedish insurance system have shown that caseworkers who are on a tight schedule might focus more on assessing the sick-listed worker’s right to receive benefits instead of focusing on their individual needs. Ståhl et al. [40] claims that there is a distinction between a correct and a good decision, where a correct decision is made in accordance with legislation while a good decision takes into account dignity, autonomy, and individual needs. They argue that it is necessary to make exceptions to rules to make good decisions [40]. In the spirit of MI, the counselor should be able to give up their expert role and support the client’s autonomy and expertise in his or her own decisions regarding change [35]. Thus, applying the MI approach when counseling sick-listed workers in a RTW process could arguably be one of these good decisions.

Work is important for an individual’s self-confidence and self-esteem [33]. In the present study, being absent from work due to sick leave led to feelings of guilt, even when being on grade sick leave. Garthwaite [41] found that the need to validate illness was important for sick-listed workers, and the search for legitimacy was a large part of their current lives. Similar to the current study, being on sick leave included a search for legitimacy. Gaining acceptance from others about their situation can make it easier for the sick-listed worker to accept their own absence from work. This is in accordance with a previous study [42], where the decision to disclose or not disclose an invisible illness was difficult and disclosing the illness could lead to both support and experiences of stigma. The acceptance and support that the participants received from the MI caseworkers in the present study helped them to reduce feelings of guilt, stigma, and perceived barriers to RTW. Self-understanding and viewing oneself as an active agent is necessary to taking control of ones RTW process [39]. Similarly, in MI the client takes an active part in his or her process of change, in this case, the RTW process.

In the MI sessions in the current study, the sick-listed workers received personal feedback about their presented RTW plan, such as adjusting their RTW pace, workload, work tasks, and working time. The role of a MI practitioner is not to provide answers and solutions to the client, but to recognize and support the client’s insights and capabilities of providing solutions to his or her own challenges [43]. Hence, when the sick-listed workers in the current study perceived RTW adjustments as positive and useful, it is based on insights and reflections from the sick-listed worker, that were elicited, reflected, and summarized by the MI caseworker. Merely discussing their situation with MI caseworkers may also result in increased awareness of the sick-listed worker’s own capacity, which, arguably, is a component of self-efficacy [44]. Norlund et al. [45] state that self-efficacy, the belief in ones’ ability to achieve a given goal or task, affects thought patterns that could be barriers to returning to work. Furthermore, receiving positive feedback from others may also increase the individual’s self-efficacy [45]. In the current study, when the MI caseworker established a supportive relationship with the sick-listed worker and gave feedback to their thoughts and insights on their RTW plan, this may have strengthened the sick-listed workers self-efficacy, which is known to increase the likelihood of RTW [11, 12].

### Strengths and limitations

A strength of the current study was the use of semi-structured interviews, which allow the participants to explain and describe their situations and experiences of the MI sessions and the RTW process. This study used a broad exploratory approach with a heterogenous sample to uncover the different experiences and nuances. Both the analysis and preliminary results were presented and discussed with all the authors to strengthen the interpretations and validate the results. Interviews were conducted from 2 to 4 months after the MI sessions, and the participants may have failed to recall information and details about their experiences. Furthermore, there is a risk that the sick-listed workers could have held back information in the MI sessions if they feared there could be consequences for their benefits. However, none of the participants expressed such barriers in the interviews. The current study recruited participants from a RCT, with a response rate of approximately 8%. From this sample, the current nested study had a response rate of 55%. This indicates a selection bias, where participants could be more motivated in general, not necessarily representing the variances in the experiences of the MI sessions. Thirteen of the sixteen recruited participants were women. However, we did not find any gender differences in terms of how they experienced the MI sessions.

## Conclusion

Sick-listed workers considered the MI sessions to be a positive experience due to the positive relationships formed with the MI caseworkers, the normalizations of sick leave that they experienced, and the help they received in adjusting their RTW strategies. Having an early face-to-face follow-up session using MI may positively affect sick-listed workers’ relationship to NAV and increase trust towards public services such as NAV. NAV caseworkers and other professionals working with individuals attempting to RTW may benefit from using MI as a method for helping sick-listed workers to RTW. However, when the sick-listed worker is not in need of information or RTW adjustments, the MI sessions were not experienced as useful. This suggest that future interventions may benefit from selecting individuals who express the need for such RTW support. Findings from this study may be transferred to other similar systems like NAV and may also be transferable to other one-on-one counseling situations.

## Supplementary information

**Additional file 1.** Interview guide. This document contains the questions used in the interviews with the participants. Translated into an english language version.

## Data Availability

To protect the anonymity of the participants, the datasets generated and analyzed during the current study are not publicly available. Redacted versions are available from the corresponding author upon reasonable request.

## Abbreviations

MI: Motivational interviewing
NAV: Norwegian Labor and Welfare Administration
RCT: Randomized controlled trial
RTW: Return to work
