# Supplementary material for: Sick-listed workers’ experiences with motivational interviewing in the return to work process: a qualitative interview study
Source: BMC Public Health. 2020 Feb 28;20:276. doi: 10.1186/s12889-020-8382-9 (PMC7048031; doi:10.1186/s12889-020-8382-9)
Supplement: Supplementary file 1 — Additional file 1. Interview guide. This document contains the questions used in the interviews with the participants. Translated into an english language version. [file 12889_2020_8382_MOESM1_ESM.docx]

**Interview guide – MI participants**

Can you start with telling me about yourself?

- Age

- What is the reason for your sick leave?

- How long have you been on sick leave?

- Current sick leave status? (in %)

- What is your line of work? How would you characterize your job?

- How many counseling sessions at NAV have you participated in?

1. **Can you tell us about your current situation as sick-listed?**
   1. What is it like being sick listed?
   2. How has your process for returning to work been?
2. **Can you tell us what kind of follow-up you have received- and from whom?**
3. **Can you tell us about the counseling sessions at NAV?**
   1. What was your overall experience from these sessions?
   2. How did you experience the relationship with the NAV caseworker?
   3. What were your expectations before going to the counseling sessions?
   4. What were your thoughts about returning to work prior to these sessions? And has your plan or thoughts changed after these sessions?
4. **What consequences have these counseling sessions had for you?**
   1. Have you made any changes based on what you talked about in the sessions?
   2. If you have not done any changes, can you elaborate?
   3. Was a plan to RTW drawn up or made in these sessions with NAV?
      1. If so, can you tell us the plan?
5. **What are your thoughts for the future?**
   1. What are your thoughts on returning to work?
   2. What makes it important for you to return to work?
   3. What are your goals or expectations for returning to work?
      1. From yourself, from others (colleagues, employer, friends, family)?
      2. Has these expectations changed after the sessions at NAV?
   4. Who (or what) is supporting you to return to work?
